# The thesis defense as an academic rite: a call for institutional standards of conduct and excellence in postgraduate programs

**DOI:** 10.1590/acb415126

**Published:** 2026-09-11

**Authors:** Diego Adão, Rafael Leite Pacheco, Leonardo Yuri Kasputis Zanini, Gabriela Caetano Lopes Martins, Mariana Xavier Tavares Sacramento, Carolina Rodrigues Novais, Leonardo de Mello Del Grande, Rachel Riera, Marcelo Moura Linhares

**Affiliations:** 1 Universidade Federal de São Paulo – Escola Paulista de Medicina – Department of Surgery – São Paulo (SP), Brazil.; 2 Hospital Sírio-Libanês – Health Technology Assessment Center – São Paulo (SP), Brazil.; 3 Centro Universitário São Camilo – São Paulo (SP), Brazil.

**Keywords:** Education, Graduate, Academic Dissertations as Topic, Educational Measurement, Organizational Culture, General Surgery

## Abstract

The public defense of a master’s dissertation or doctoral thesis is a defining moment in postgraduate education. It represents both an academic evaluation and a ceremony that marks the transition of a researcher into an independent scientific career. Yet in many postgraduate programs, the conduct expected of candidates, examiners, and the audience during these proceedings remains largely unwritten, transmitted informally from one generation to the next. We argue that this absence of codified standards carries real consequences for the quality and fairness of the evaluation process. Drawing on the experience of a Brazilian surgical postgraduate program and on the international literature on doctoral defense formats, we propose that institutions should develop explicit, written guidelines covering the responsibilities of all participants in the defense ceremony. Such guidelines would strengthen the evaluative rigor of the defense, protect candidates from avoidable failures of process, and reinforce the institutional culture of academic excellence that distinguishes high-quality postgraduate programs.

## The defense as an academic rite of passage

The public defense of a doctoral thesis or master’s dissertation occupies a singular position in postgraduate education. It is the moment in which a candidate’s years of training, research, and writing converge into a unique, time-limited, public performance. Across cultures and disciplines, this event is recognized as both an examination and a rite of passage^1^. Lantsoght^1^, in a study analyzing defense formats across 26 countries, identified four structural elements that vary internationally: the timing of the defense relative to thesis publication, the number of steps in the process, whether the event is public or private, and the internal timeline of the ceremony itself. Despite this diversity of formats, one feature is nearly universal: the defense is a solemn act through which the academic community evaluates not only the written work but also the candidate’s capacity to reason critically, to argue under pressure, and to demonstrate genuine mastery of the subject.

In the university hospital postgraduate programs, this ceremony acquires additional weight. The candidate is expected to defend methodological choices that may have direct implications for patient care. In surgical programs, for instance, the examiners are often surgeons whose own clinical and scientific authority is on display. The audience may include residents, fellows, and junior faculty who will face this moment in the future. What happens during a defense, and how it happens, therefore shapes the academic culture of the entire program.

Yet in many programs, the behavioral expectations for this event remain implicit. Candidates learn how to prepare themselves by watching others or by receiving informal advice from their advisors. Examiners bring their own habits, sometimes constructive, sometimes not. The audience is left without guidance. The result is considerable variability in the quality, fairness, and solemnity of defenses in the same institution. We believe this variability is not inevitable. It reflects, instead, the absence of a resource that most programs have never thought to create: a written code of conduct for the defense ceremony.

## The candidate: preparation, posture, and the art of defending

Preparation for the defense begins long before the day of the ceremony. The candidate should re-read the entire thesis, annotate its potential weaknesses, and anticipate the lines of questioning that each examiner is likely to pursue. Knowing the academic profile and recent publications of each committee member is not a courtesy; it is a tactical necessity. Lantsoght’s^2^ survey of 204 doctoral candidates across multiple countries found a significant positive correlation between the degree of preparation and the candidate’s own perception of the defense experience. Candidates who rehearsed their presentations, practiced answering questions, and familiarized themselves with committee members’ expertise reported higher satisfaction and lower anxiety.

The composition of the examining committee is a decision that the advisor and the candidate should make together, deliberately. The guiding criterion is straightforward: each member should be selected for scientific competence and familiarity with the topic under examination, not for personal affinity or the expectation of a lenient evaluation. The temptation to assemble a *friendly* committee often stems from the anxiety about criticism. That anxiety, however, is better channeled into strengthening the thesis itself. A committee composed of rigorous, knowledgeable examiners will expose genuine weaknesses in the work and, in doing so, improve the final product far more than a committee chosen for its willingness to approve without scrutiny.

These criteria align with the indirect quality indicators used by the evaluation system managed by the Coordenação de Aperfeiçoamento de Pessoal de Nível Superior (CAPES), which considers committee composition itself as a marker of dissertation and thesis quality. Among the criteria CAPES recommends, there are: that members hold an academic degree compatible with the evaluation being conducted; that they act with impartiality, avoiding conflicts of interest; and that the composition respect principles of exogeneity—*i.e.*, the inclusion of examiners external to the graduate program, to the institution, and, when possible, to the immediate research group.

Beyond the immediate evaluation, the selection of committee members is also a strategic academic act. Examiners who are recognized authorities in their fields can become future collaborators, provide access to professional networks, and lend credibility to the candidate’s early career. A well-chosen committee may open doors that the candidate will walk through for years to come.

The invitation to committee members should be extended at least 60 days before the scheduled defense, accompanied by the offer of a printed copy of the thesis, so that examiners have adequate time to read the work in its entirety and prepare a substantive examination. This advance notice is not merely a bureaucratic formality. It is the minimum condition for ensuring that each examiner arrives at the defense with the depth of familiarity that a rigorous evaluation demands.

On the day itself, practical details matter more than candidates typically realize. Arriving early to test audiovisual equipment, bringing a printed copy of the thesis for reference during questioning, and having pen and paper to organize responses to each examiner’s points are small measures that signal professionalism and prevent unnecessary disruptions. Sleep, nutrition, and appropriate attire are not trivial recommendations; they reflect the candidate’s respect for the solemnity of the event and for the time invested by the committee.

The most consequential aspect of the candidate’s performance, however, is neither the presentation nor the logistics. It is the quality of the defense itself, in the literal sense of the word. A defense is an exercise in reasoned argumentation. The candidate who agrees passively with every criticism, regardless of its merit, fails to demonstrate the intellectual independence that the degree is meant to certify. Conversely, the candidate who resists every point is not defending but obstructing. The appropriate stance is one of calibrated assertiveness, balancing intellectual independence with openness to valid critique. When the evidence supports the candidate’s position, it should be stated; when a limitation is real, acknowledging it with honesty and precision is a mark of scientific maturity, not weakness.

Formality during the proceedings also deserves explicit attention. Personal acknowledgments, references to friendships with committee members, and emotional tributes are natural and welcome, but they belong in the closing remarks after the committee’s deliberation, not during the examination itself. Mixing personal affect with academic evaluation compromises the rigor of both. The appropriate sequence is clear: first, the scientific exchange; then, after the committee announces its decision, the personal expressions of gratitude. This distinction, simple as it may seem, is frequently blurred in practice, and its enforcement depends on establishing the expectation in advance.

After the defense, the candidate’s obligations do not end with the committee’s approval. Sending individual messages of thanks to each examiner within 48 hours is a professional gesture that reinforces the collegial nature of academic relationships. More substantively, the candidate must address all corrections requested by the committee within the regulatory deadline. A defense that results in approval with corrections is the standard outcome. What matters is the diligence with which those corrections are implemented and the intellectual honesty with which the candidate incorporates the committee’s feedback into the final version of the work.

## The examining committee: evaluation, not spectacle

If the candidate carries the burden of demonstrating competence, the examining committee carries the equally demanding burden of ensuring that the evaluation is fair, substantive, and rigorous. This responsibility begins well before the defense. Reading the thesis in its entirety and with sufficient advance notice is a non-negotiable prerequisite. A committee member who arrives having skimmed the abstract and browsed a few tables cannot produce the kind of specific, informed questioning that the ceremony demands.

The quality of the examination depends on the quality of the questions. Examiners should arrive with a prepared line of questioning, not merely opinions. Each question should serve one of three functions: testing the candidate’s mastery of the subject, probing the robustness of the methodology, or assessing the candidate’s capacity for critical reasoning. Generic questions, questions generated by artificial intelligence tools without critical appraisal, or questions that stray far from the scope of the work fail all three criteria. Even when an examiner fully agrees with the thesis, it remains the examiner’s duty to formulate questions that challenge the candidate. A committee that only praises does not fulfill its evaluative function.

Although it is advisable to prepare some questions in advance, this preparation should not be mistaken for a rigid script. A defense is a dynamic and inherently unpredictable exchange, and some of the most productive lines of inquiry emerge organically from the candidate’s own answers rather than from anything planned beforehand. Examiners should therefore remain open to following wherever the dialogue leads, using their prepared questions as a foundation rather than a constraint.

One practice that deserves particular attention, because it recurs with some frequency, is the transformation of the oral examination into a line-by-line text review. Pointing out typographical errors, formatting inconsistencies, or page numbers during the public session is not an examination of the candidate’s scientific competence. It is a copy-editing exercise performed in the wrong setting. Minor corrections should be compiled in writing and delivered to the candidate after the ceremony. The public defense should remain focused on ideas, methods, and evidence, not on punctuation.

The committee’s conduct also sets the emotional tone of the event. Research on doctoral candidates’ perceptions has consistently shown that the power imbalance between examiners and candidates creates vulnerability^1,3^. Morley et al.^3^ studied the quality and fairness of British PhD assessments and found that committee dynamics could either support or undermine the candidate’s ability to perform. Firmness and rigor in questioning are entirely compatible with respect. Humiliation is a failure of professionalism.

The committee chair, typically the candidate’s advisor, has a distinct role. The chair conducts the session, controls time, introduces the participants, and ensures that the candidate receives a fair opportunity to respond to each examiner. Critically, the chair must not answer on behalf of the candidate. Doing so, even with the best of intentions, undermines the very autonomy that the defense is designed to assess. The chair also sets the ceremonial tone, opening and closing the session with the appropriate formulas, maintaining decorum without excessive rigidity, and managing the transition between the public evaluation and the private deliberation. When a chair performs these functions well, the defense flows naturally. When the chair is absent or passive, the ceremony loses its structure and its meaning.

External examiners, invited from other institutions, face an additional set of expectations. They must confirm their participation well in advance, arrive punctually, and familiarize themselves with the regulations and customs of the host program, a task that would be greatly facilitated by the existence of written institutional guidelines on defense conduct, such as the one this article proposes. Their examination should reflect the same depth of preparation as that of internal members. An external examiner who reads superficially or who asks questions unrelated to the thesis does a disservice not only to the candidate but to the interinstitutional relationship that the invitation was meant to cultivate.

## The audience and the institutional dimension

The audience at a thesis defense is not a passive spectator. Its behavior contributes directly to the atmosphere of the ceremony. Silence during the presentation and examination, discreet movement, and applause only at appropriate moments (after the presentation and after the committee’s announcement of the result) are expectations that may seem self-evident but are, in practice, frequently violated. In programs in which defenses are well attended by residents and junior faculty, the ceremony also serves a formative function. It models the standards of academic discourse to which future candidates will be held. A disorderly defense teaches disorder.

This brings us to the institutional dimension of the argument. The quality of a postgraduate program is reflected not only in the impact of its publications or the rigor of its curricula, but also in the culture that surrounds its most visible academic rituals. In Brazil, the evaluation system managed by CAPES considers, among many indicators, the quality of dissertations and theses produced by each postgraduate program. A well-conducted defense is the final assurance that the candidate’s work meets the program’s standards and that the evaluation process itself was carried out with integrity.

Institutional formatting guidelines, such as those published by the Universidade Federal de São Paulo for its postgraduate programs^4^, standardize the written product. We argue that an analogous effort is needed for the oral evaluation. This effort also resonates with the broader movement toward open and responsible research practices. Just as open science initiatives seek to make the processes of knowledge production more transparent, reproducible, accountable, codifying the conduct expected during a thesis defense makes explicit and shareable a process that has traditionally relied on tacit, informally transmitted norms.

Written guidelines on defense conduct serve multiple functions: they protect candidates by making expectations transparent, they hold examiners accountable to a shared standard of quality, and they preserve the solemnity and academic character of the event across generations of faculty and students.

These expectations apply equally when the defense is conducted in a virtual format. Remote participation does not reduce the formality of the event, transferring that formality to a different medium. All participants, whether candidates, examiners, or audience members, should keep their cameras on throughout the session, ensure adequate lighting and a quiet environment free from interruptions, maintain a stable internet connection, and have a fully charged device available as a precaution against power failures. Backgrounds should be neutral and uncluttered, and microphones should remain muted except when the participant is speaking, so that ambient noise does not compromise the solemnity or the intelligibility of the proceedings.

## A practical proposal

Based on our experience coordinating the Postgraduate Program in Interdisciplinary Surgical Science at the Universidade Federal de São Paulo and informed by the international literature on doctoral defense formats and preparation^1,2,5^, we developed a written internal guide of etiquette and best practices for thesis defenses^6^. The guide addresses, in explicit terms, the responsibilities of the candidate (from preparation weeks before the defense to post-defense follow-up), the responsibilities of each committee member (including the chair and external examiners), and the expected conduct of the audience. It covers practical details such as attire, equipment testing, time management, use of formal forms of address, and the appropriate moment for personal acknowledgments. It also articulates principles that are rarely stated but widely assumed: that the examiner’s role is to evaluate, not to humiliate; that the candidate’s role is to defend, not merely to agree; and that the audience’s role is to witness an academic act, not to disrupt it.

We do not claim that our guide is the only possible model, or that it should be adopted without adaptation by other programs. Institutional contexts differ, and what is appropriate at one university may require adjustment at another. What we do argue, with conviction, is that the exercise itself is valuable. The act of writing down what a program expects from its defenses forces a conversation that many departments have never had. It reveals implicit assumptions, resolves ambiguities, and produces a document that can be shared with candidates and committee members alike, well before the defense takes place.

Programs that aspire to the highest CAPES evaluations, or that seek international recognition for the quality of their training, cannot afford to leave the conduct of their most important academic ceremony to chance. A defense should be, at once, a rigorous evaluation and a celebration of the knowledge produced. Achieving both requires intentionality, and intentionality, in an institution, requires written—and shared—standards.

## Conclusion

Codifying the behavioral expectations for thesis defenses may strengthen the evaluative rigor and institutional culture of postgraduate programs. Written guidelines protect candidates, hold examiners accountable, and signal to the academic community that the program takes its most visible ritual as seriously as its research output.

## Data Availability

Data sharing is not applicable.
